# Supplementary figures and images for: Loss of Pol III repressor Maf1 in neurons promotes longevity by preventing the age-related decline in 5S rRNA and translation
Source: PLoS Biol. 2025 Jul 15;23(7):e3003250. doi: 10.1371/journal.pbio.3003250 (PMC12262857; doi:10.1371/journal.pbio.3003250)

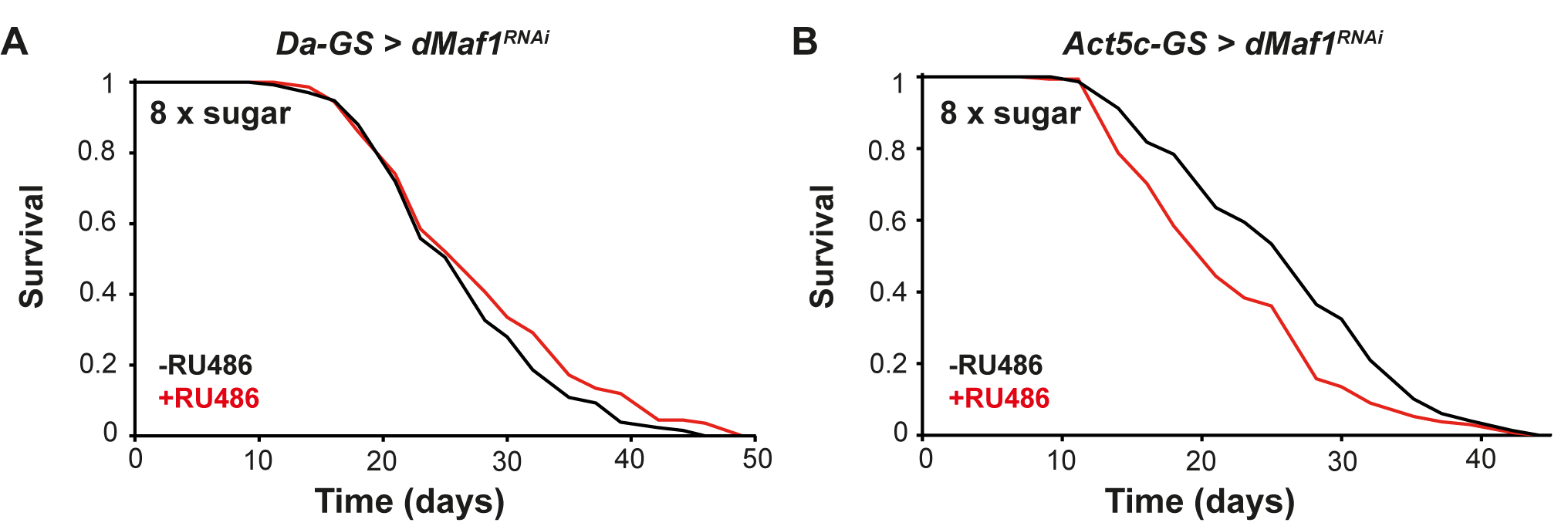

Supplement: S1 Fig — A, Lifespan of females with adult-specific, ubiquitous induction of dMaf1RNAi(V109142) driven by Da-GS fed a high sugar diet (−RU486: n = 130/20, +RU486: n = 138/12, p = 0.0652, log-rank test). B, Lifespan of females with adult-specific, ubiquitous induction of dMaf1RNAi(V109142) driven by Act5C-GS fed a high sugar diet (−RU486: n = 148/2, +RU486: n = 136/14, p = 3 × 10^−4^, log-rank test). These experiments were done at the same time as lifespans in Fig 1. Data underlying the graphs in this figure can be found in S1 Data. (TIF) [file pbio.3003250.s001.tif]

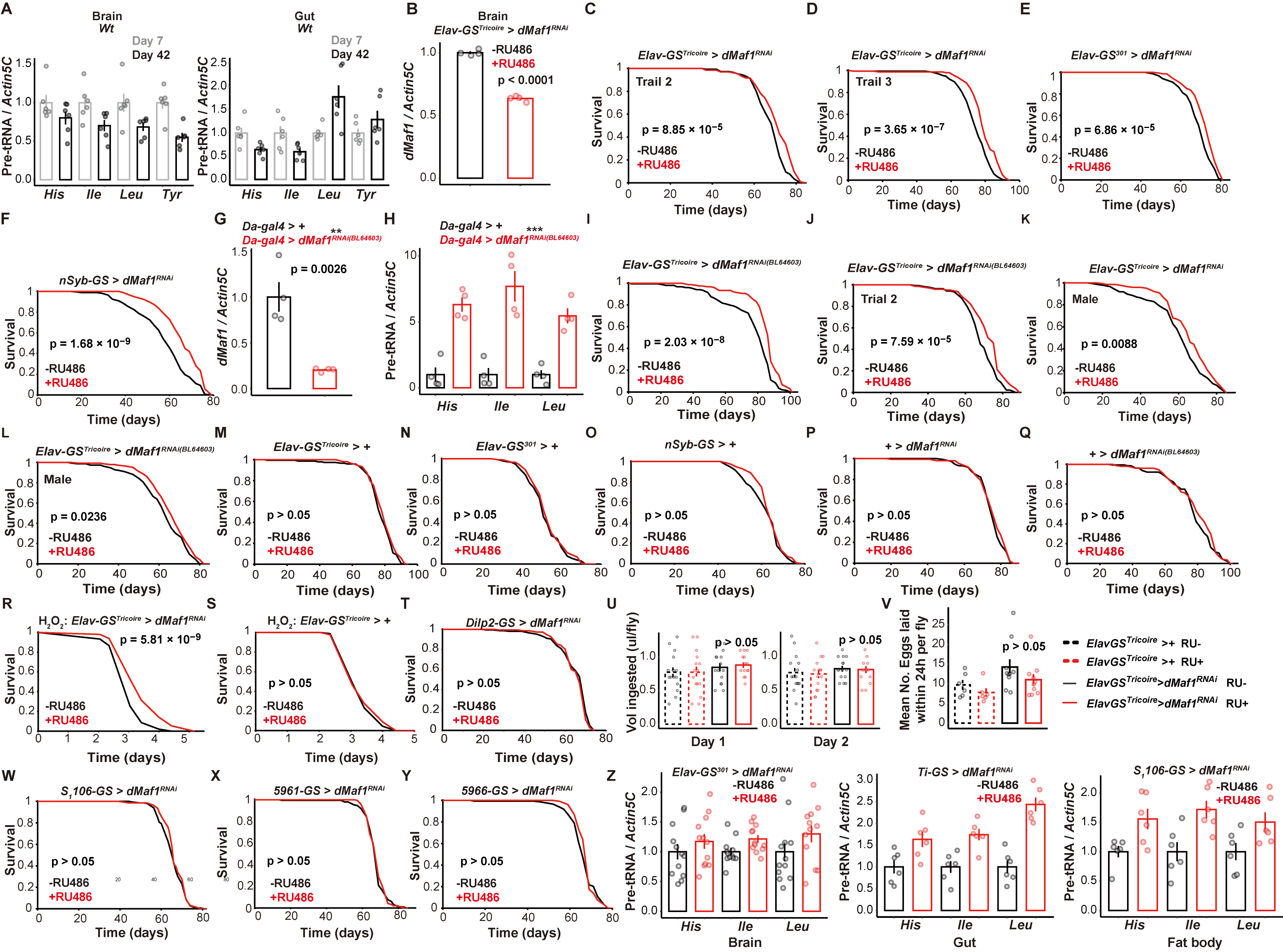

Supplement: S2 Fig — A, qPCR quantifications normalized by Actin5C mRNA expression of pre-tRNAs in female brains (n = 6 biologically independent samples, age effect, p < 1 × 10^−4^, pre-tRNAs effect, p = 0.7430, age-by-pre-tRNAs interaction, p = 0.5198, LM), or guts (n = 6 biologically independent samples, age effect, p = 0.4169, pre-tRNAs effect, p < 1 × 10^−4^, age-by-pre-tRNAs interaction, p < 1 × 10^−4^, LM) from same wild-type flies with 7- and 42-days’ age. B, qPCR quantification of dMaf1 mRNA in female brains after RU486 induction of dMaf1RNAi(V109142) under Elav-GSTricoire (n = 4 biologically independent samples, p < 1 × 10^−4^, Student t test). C, D, The second trail of the lifespan assay (−RU486: n = 147/2, +RU486: n = 132/1, p = 8.85 × 10^−5^, log-rank test), and the third trail (−RU486: n = 154/2, +RU486: n = 132/17, p = 3.65 × 10^−7^, log-rank test) on flies with adult-specific, pan-neuronal induction of dMaf1RNAi(V109142) driven by Elav-GSTricoire. E, Lifespan of females with adult-specific, pan-neuronal induction of dMaf1RNAi(V109142) driven by Elav-GS301 (−RU486: n = 151/3, +RU486: n = 142/6, p = 6.86 × 10^−5^, log-rank test). F, Lifespan of females with adult-specific, pan-neuronal induction of dMaf1RNAi(V109142) driven by nSyb-GS (−RU486: n = 174/0, +RU486: n = 172/2, p = 1.68 × 10^−9^, log-rank test). G, qPCR quantification of dMaf1 mRNA (n = 4 biologically independent samples, p = 0.0026, Student t test) or H, pre-tRNAs (n = 4 biologically independent samples, genotype effect, p < 1 × 10^−4^, pre-tRNAs effect, p = 0.2546, genotype-by-pre-tRNAs interaction, p = 0.2546, LM) in female flies expressing dMaf1RNAi(BL64603). I, The first trial of the lifespan assay (−RU486: n = 149/4, +RU486: n = 139/12, p = 2.03 × 10^−8^, log-rank test), and J, the second trial (−RU486: n = 138/0, +RU486: n = 141/0, p = 7.59 × 10^−5^, log-rank test) of females with adult-specific, pan-neuronal induction of dMaf1RNAi(BL64603) driven by Elav-GSTricoire. K, Lifespan of males with adult-specific, pan-neuronal induc [file pbio.3003250.s002.tif]

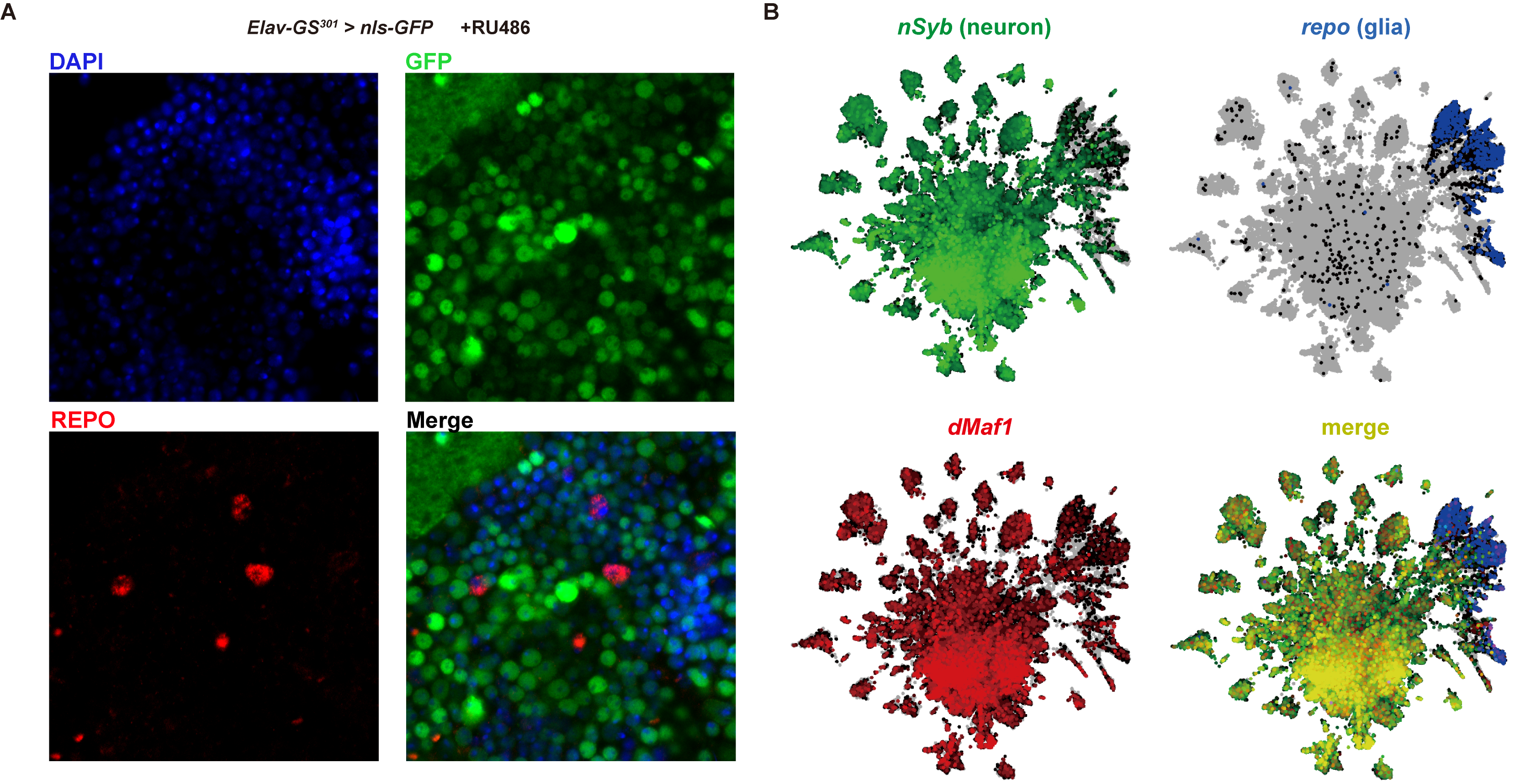

Supplement: S3 Fig — A, Images from the cell body layer of the central brain show ElavGS + RU486 induced nls-GFP expression in REPO-negative (neuronal) cells. B, Images from the SCope database of adult Drosophila brain reveal dMaf1 mRNA is highly expressed within neurons but lowly within glia. (TIF) [file pbio.3003250.s003.tif]

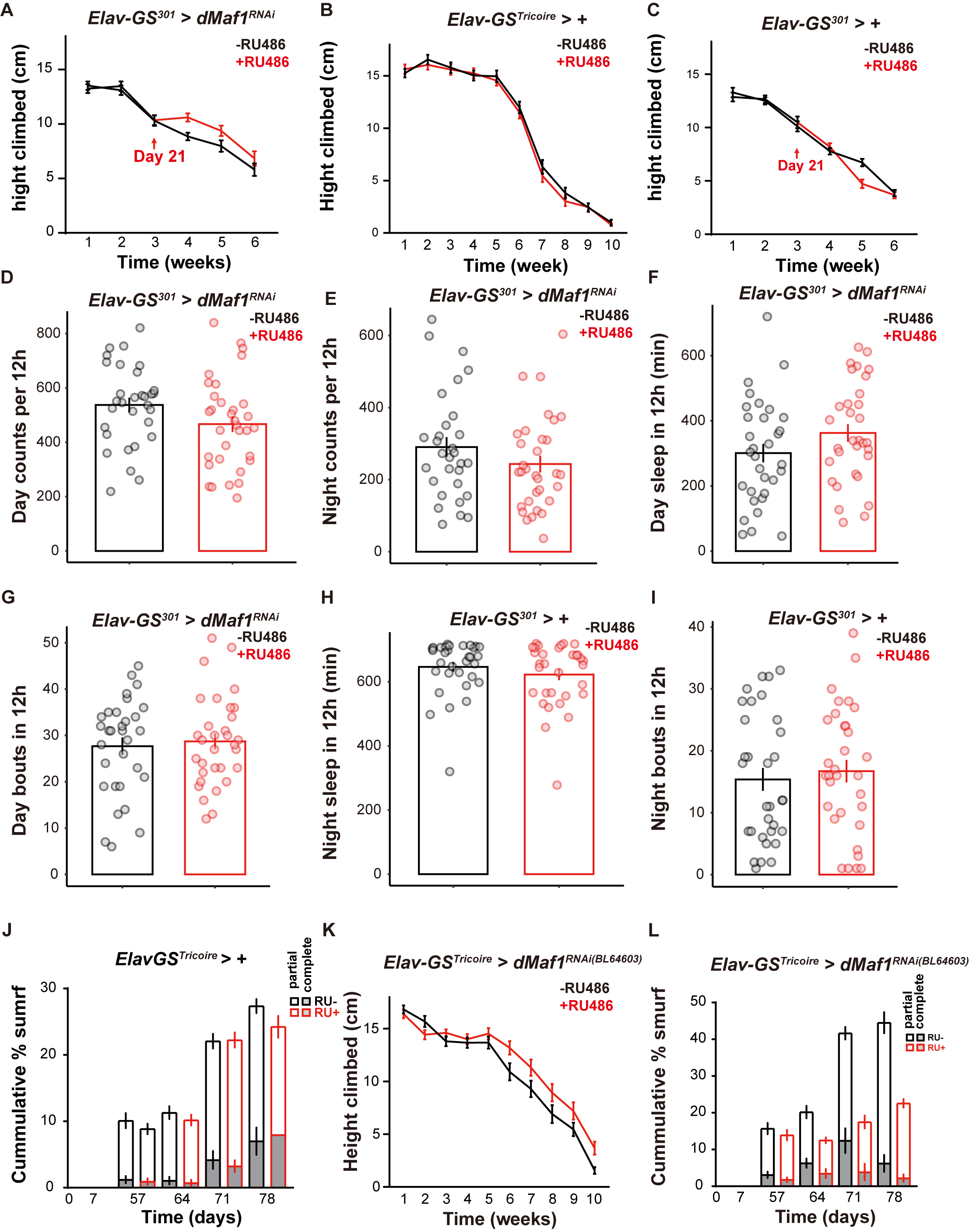

Supplement: S4 Fig — A, Height climbed during negative geotaxis assays by female flies with a later-life (from day 21), pan-neuronal induction of dMaf1RNAi(V109142) driven by Elav-GS301 (n = 50–60 flies, RU486 effect, p = 0.0011, age effect, p < 1 × 10^−4^, RU486-by-age interaction, p = 0.4625, LM). B, Height climbed during negative geotaxis assays by females with adult-specific, pan-neuronal induction of Elav-GSTricoire driver alone (n = 43–69 flies, RU486 effect, p = 0.2044, age effect, p < 1 × 10^−4^, RU486-by-age interaction, p = 0.9495, LM), and C, later-life (from day 21), pan-neuronal induction of Elav-GS301 driver alone (n = 43–69 flies, RU486 effect, p = 0.1536, age effect, p < 1 × 10^−4^, RU486-by-age interaction, p = 0.1020, LM). D, Quantification of day activity (n = 32 individual flies, RU486 effect p = 0.0788, Student t test), E, night activity (n = 32 individual flies, RU486 effect, p = 0.1911, Student t test), F, day sleep (n = 32 individual flies, RU486 effect, p = 0.1201, Student t test), and G, day bouts (n = 32 individual flies, RU486 effect, p = 0.6826, Student t test) of female flies with adult-specific, pan-neuronal induction of dMaf1RNAi(V109142) driven by Elav-GS301. H, Quantification of night sleep (n = 32 individual flies, RU486 effect, p = 0.3018, Student t test) and I, night bouts (n = 32 individual flies, RU486 effect, p = 0.6056, Student t test) of female flies carrying Elav-GS301 driver alone. J, Cumulative proportion of partial and complete smurfs in female flies with carrying Elav-GSTricoire driver alone (n = 170–340 flies, RU486 effect, p = 0.8758, age effect, p < 1 × 10^−4^, RU486-by-age interaction, p = 0.9229, ordinal logistic regression). K, Height climbed during negative geotaxis assays by female flies with adult-specific, pan-neuronal induction of dMaf1RNAi (BL64603) driven by Elav-GSTricoire (n = 32–72 flies, RU486 effect, p = 3 × 10^−4^, age effect, p < 1 × 10^−4^, RU486-by-age interaction, p = 0.0413, LM). L, Cumulative proportion of partial and complete [file pbio.3003250.s004.tif]

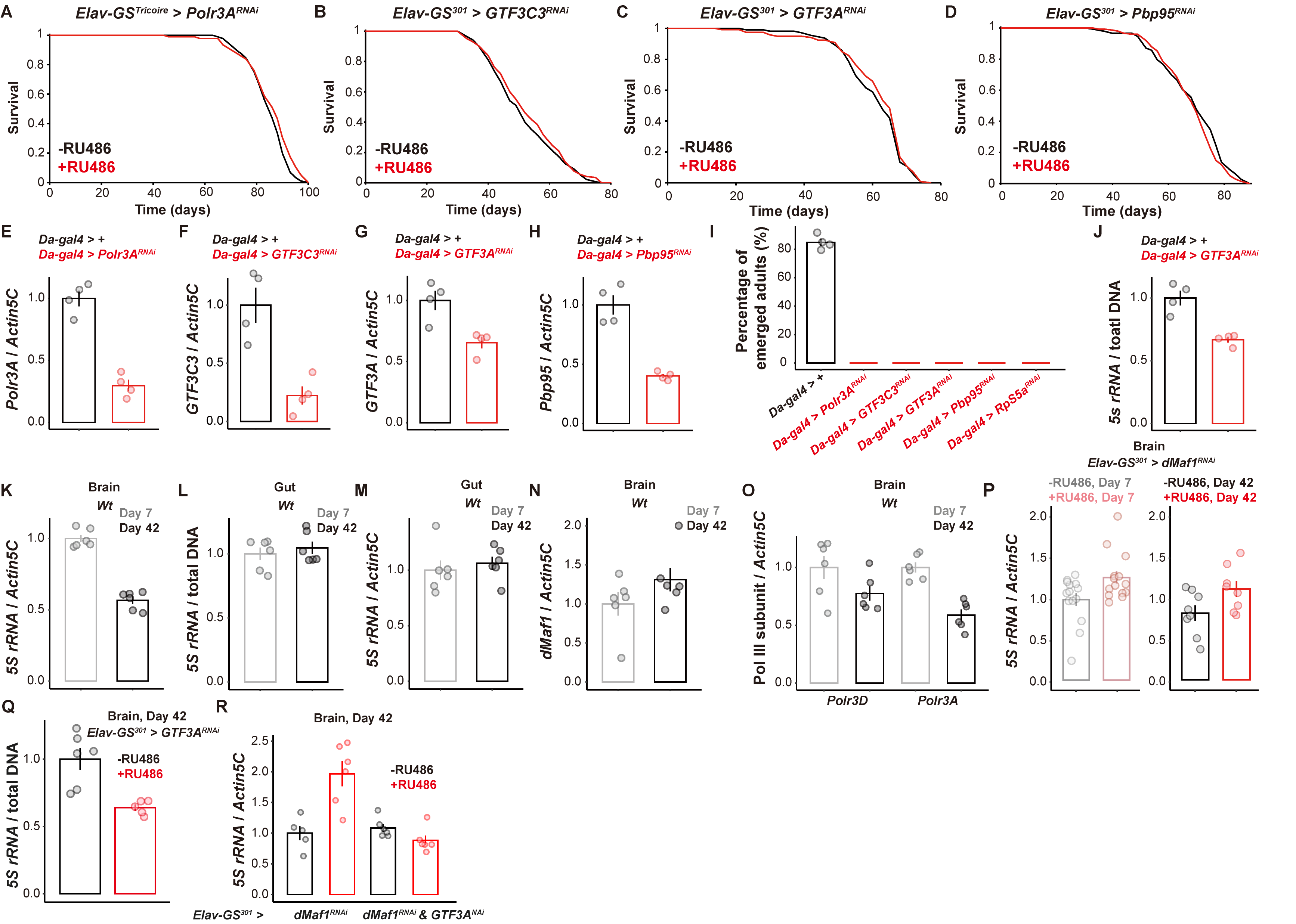

Supplement: S5 Fig — A, Lifespan of females with adult-specific, pan-neuronal induction of Polr3ARNAi driven by Elav-GSTricoire (−RU486: n = 88/1, +RU486: n = 92/0, p = 0.0606, log-rank test). B, Lifespan of females with adult-specific, pan-neuronal induction of GTF3C3RNAi driven by Elav-GS301 (−RU486: n = 134/9, +RU486: n = 143/0, p = 0.3147, log-rank test). C, Lifespan of females with adult-specific, pan-neuronal induction of GTF3ARNAi driven by Elav-GS301 (−RU486: n = 112/0, +RU486: n = 120/0, p = 0.3708, log-rank test). D, Lifespan of females with adult-specific, pan-neuronal induction of Pbp95RNAi driven by Elav-GS301 (−RU486: n = 147/0, +RU486: n = 146/4, p = 0.2031, log-rank test). E, qPCR quantifications of Polr3A mRNA normalized to Actin5C (n = 4 biologically independent samples, p = 0.0001, Student t test). F, qPCR quantifications of GTF3C3 mRNA normalized to Actin5C (n = 4 biologically independent samples, p = 0.0037, Student t test). G, qPCR quantifications of GTF3A mRNA normalized to Actin5C (n = 4 biologically independent samples, p = 0.0096, Student t test). H, qPCR quantifications of Pbp95 mRNA normalized to Actin5C (n = 4 biologically independent samples, p = 0.0004, Student t test). E–H mRNA was obtained from 3-day old larvae of the indicated genotype. I, Percentage of adults emerged after driving Polr3A, GTF3C3, GTF3A, Pbp95, or RpS5a RNAi with Da-gal4 compared to driver alone. J, qPCR quantifications of 5S rRNA normalized to total DNA (n = 4 biologically independent samples, p = 0.0018, Student t test) on 3-day old larvae of the indicated genotype. K, qPCR quantifications of 5S rRNA in female brains normalized to Actin5C mRNA (n = 6 biologically independent samples, age effect, p < 1 × 10^−4^, Student t test) and L, guts normalized either to total DNA (n = 6 biologically independent samples, age effect, p = 0.5137, Student t test) or M, Actin5C (n = 6 biologically independent samples, age effect, p = 0.5137, Student t test) from same wild-type flies. N, qPCR quantifica [file pbio.3003250.s005.tif]

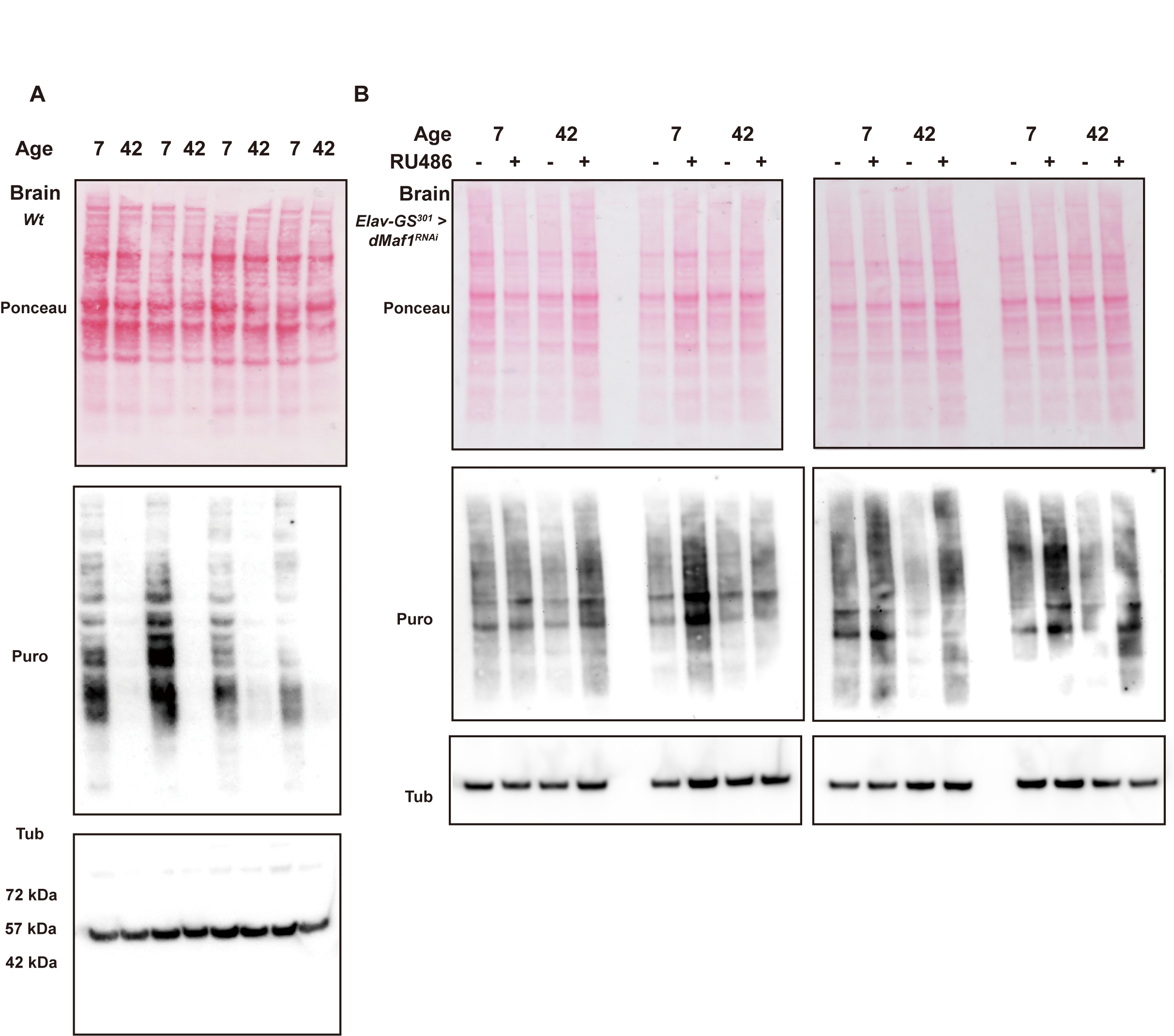

Supplement: S6 Fig — A, Ponceau S staining and western blots quantifying puromycin incorporation in female brains of wild-type flies at days 7 and 42. B, Ponceau S staining and western blots of puromycin incorporation in female brains of flies with adult-specific, pan-neuronal induction of dMaf1RNAi(V109142) driven by Elav-GS301 at days 7 and 42. In B, flies of different ages were assayed at the same time. (TIF) [file pbio.3003250.s006.tif]

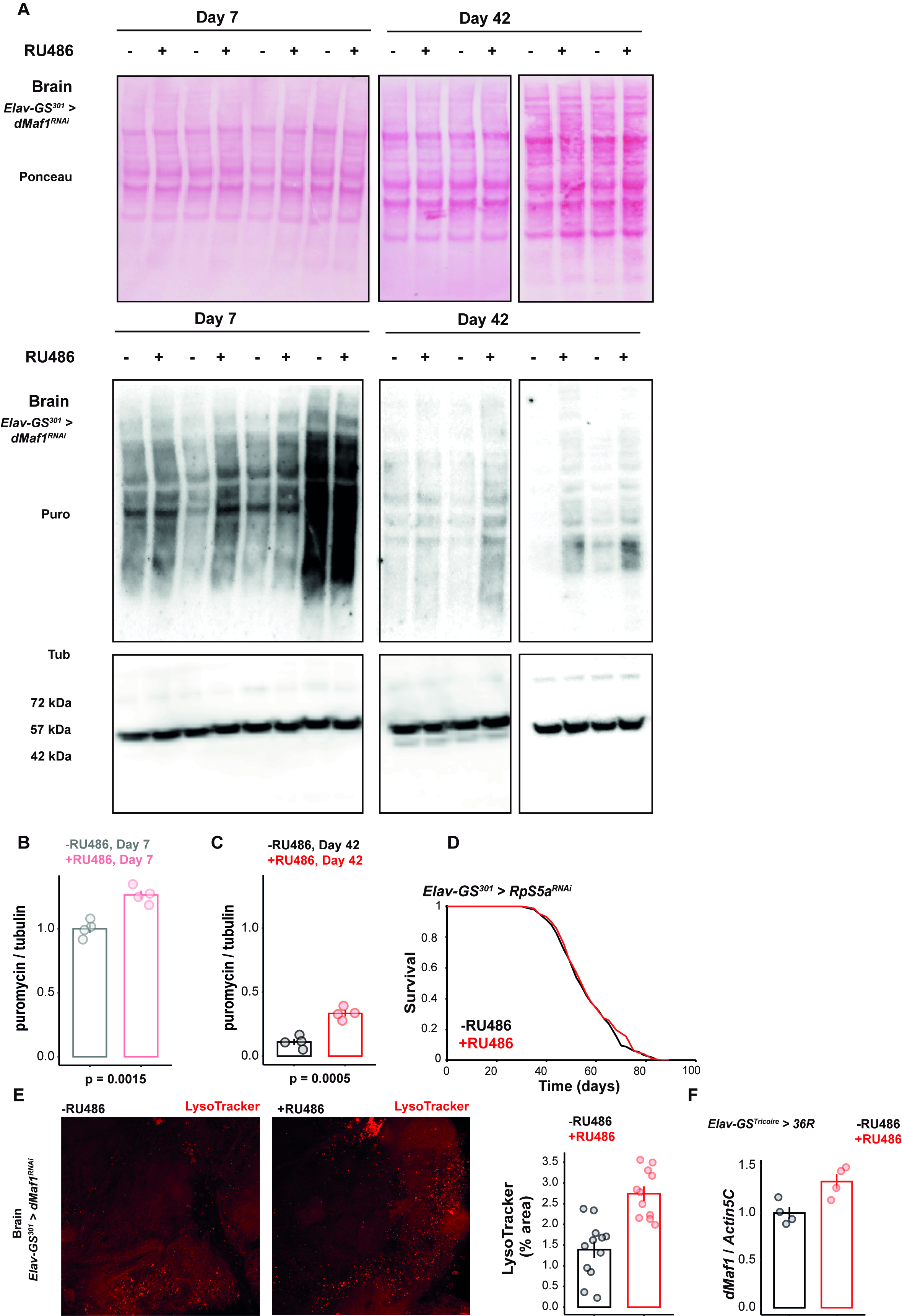

Supplement: S7 Fig — A, Ponceau S staining and western blots quantifying puromycin incorporation in female brains of flies with adult-specific, pan-neuronal induction of dMaf1RNAi(V109142) driven by Elav-GS301 at days 7 and 42. The same cohort of flies was followed though time. B, Quantification of puromycin incorporation at 7 days (n = 4 biologically independent samples, p = 0.0015, Student t test). C, Quantification of puromycin incorporation at 42 days age (n = 4 biologically independent samples, p = 5 × 10^−4^, Student t test). D, Lifespan of females with adult-specific, pan-neuronal induction of RpS5aRNAi driven by Elav-GS301 (−RU486: n = 148/1, +RU486: n = 145/3, p = 0.4988, log-rank test). E, Images from the cell body layer of the female central brain show lysosomes after RU486 induction of dMaf1RNAi(V109142) under Elav-GS301 (n = 12 biologically independent samples, p < 1 × 10^−4^, Student t test). F, qPCR quantification of dMaf1 mRNA in female heads after RU486 induction of 36R under Elav-GSTricoire (n = 4 biologically independent samples, p = 0.0168, Student t test). Data underlying the graphs in this figure can be found in S1 Data. (TIF) [file pbio.3003250.s007.tif]

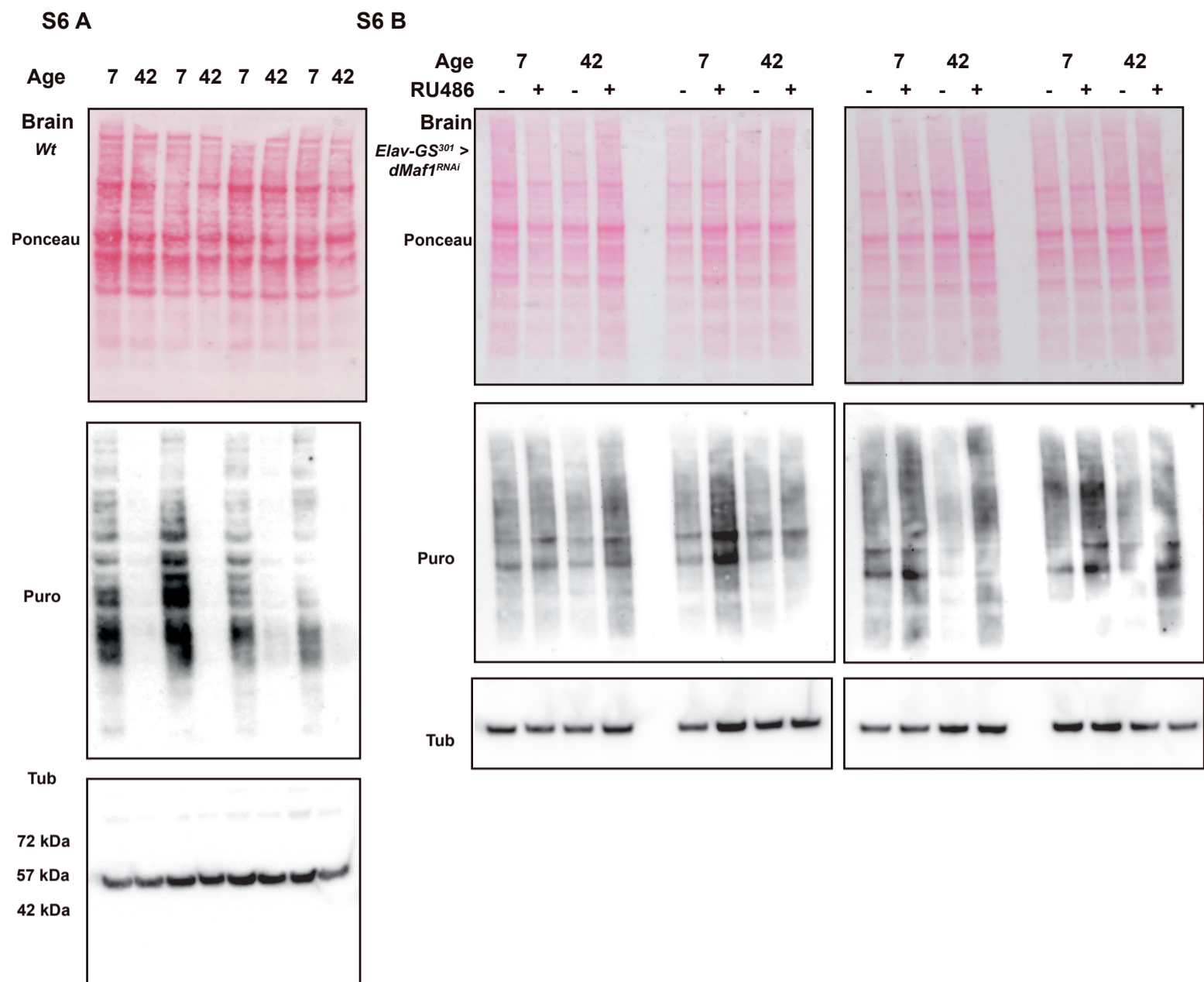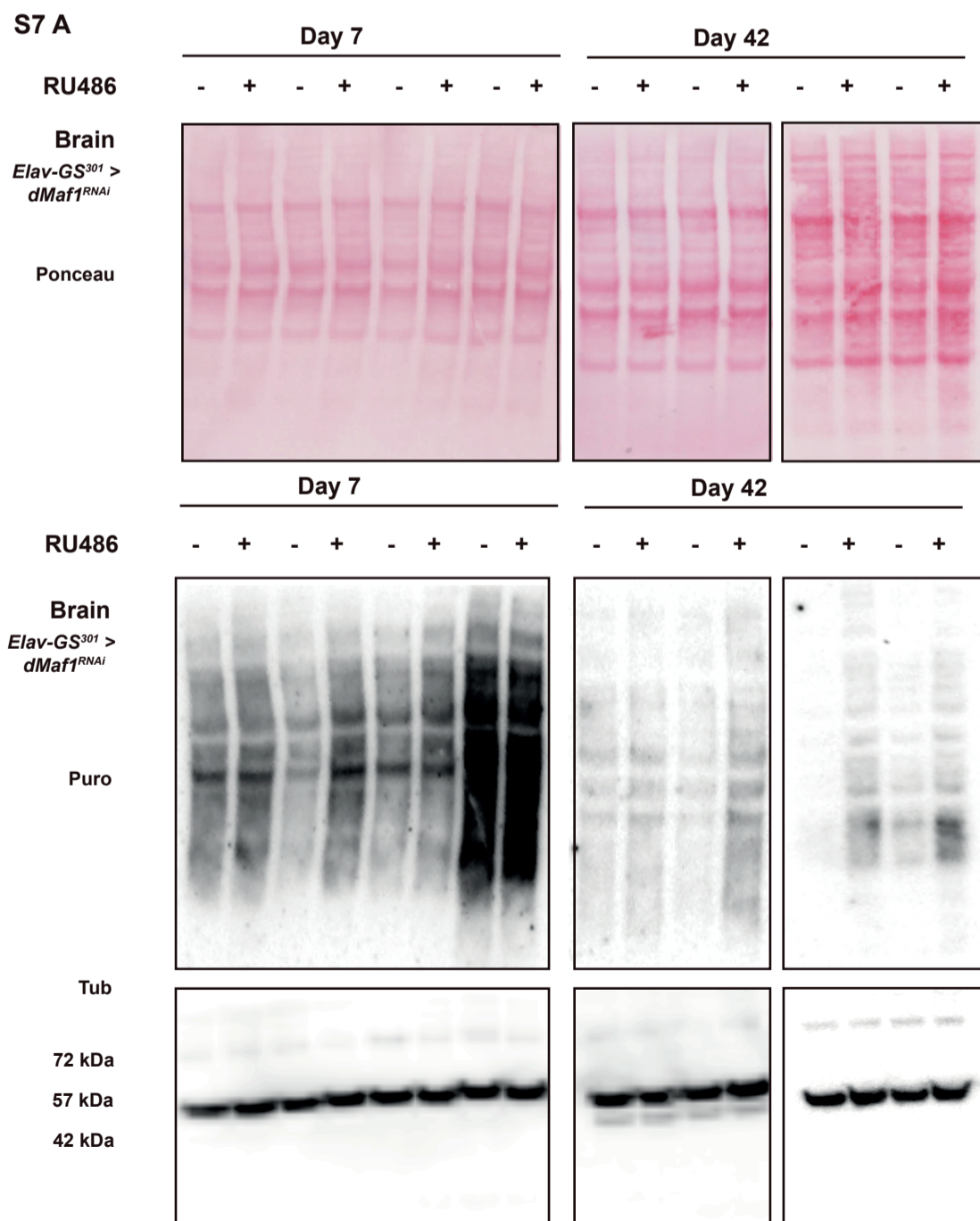

Supplement: S1 Raw Images — (PDF) [file pbio.3003250.s009.pdf]
